# Dairy Intake and the Risk of Esophageal Cancer: The JACC Study

**DOI:** 10.2188/jea.JE20220037

**Published:** 2022-06-05

**Authors:** Ahmed Arafa, Ehab S. Eshak, Kokoro Shirai, Isao Muraki, Akiko Tamakoshi, Hiroyasu Iso

**Affiliations:** 1 Public Health, Department of Social Medicine, Osaka University Graduate School of Medicine, Osaka, Japan; 2 Department of Public Health, Faculty of Medicine, Beni-Suef University, Beni-Suef, Egypt; 3 Department of Public Health, Faculty of Medicine, Minia University, El-Minia, Egypt; 4 Advanced Clinical Epidemiology, Medical Data Science, Osaka University Graduate School of Medicine, Osaka, Japan; 5 Department of Public Health, Graduate School of Medicine, Hokkaido University, Sapporo, Japan

**Keywords:** milk, cheese, yogurt, esophageal cancer, Japan

**Dear editor,**

Esophageal cancer (EC) is a common cancer, especially among Asians, and represents the 6^th^ leading cause of global cancer deaths.^1^ In Japan, 25,920 incident EC cases were diagnosed in 2018 and 11,619 EC deaths were reported in the following year.^2^ The prevalence of EC in the country is 20.5/100,000 population, the mortality rate is 9.4/100,000 population, and the 5-year relative survival rate is 41.5%.^2^ Given its heavy burden and poor prognosis, investigating the risk factors for EC is essential.

In addition to traditional risk factors for EC, such as smoking and alcohol intake,^1^ accumulating epidemiological evidence has suggested that some dietary factors could be related to the risk of EC; for example, a lower risk among vegetable consumers and a higher risk among meat consumers. Still, the role of dairy products in the development of EC is far from being decided.^3^^,^^4^

Dairy products are rich sources of many vitamins and minerals, such as vitamin D and calcium, that have anticarcinogenic effects through their antioxidative and anti-inflammatory roles. They also include probiotics that protect from cancer via preserving homeostasis, enhancing the host’s immune response, downregulating uptakes of several carcinogens, and inducing antioxidative and antiproliferative functions.^5^^,^^6^

Herein, we used the data of a large prospective cohort study, the Japan Collaborative Cohort (JACC) Study, to detect the association between the intakes of three dairy products (milk, cheese, and yogurt) and the risk of EC incidence among Japanese.

The Research Ethics Committees of Nagoya University School of Medicine and Osaka University approved the protocol of the JACC Study. During the period between 1988 and 1990, a total of 110,585 people, aged 40–79 years, were recruited from 45 areas across Japan to participate in the JACC Study. Participants responded to a baseline self-administered questionnaire assessing several lifestyle characteristics and the intakes of common foods and beverages.^7^ In the current study, we excluded people with a positive history of cancer before baseline, people with no cancer follow-up, and people who did not report their dairy intake. Eventually, the following number of participants were analyzed according to their responses to the questions about dairy intake: milk (*n* = 58,656), cheese (*n* = 49,302), and yogurt (*n* = 49,934).

Using hospital and population-based registries or death certificates, EC incidence was reported per the International Classification of Diseases (code C15) in 24 areas during the follow-up period between 1988 and 2009. A self-administered food frequency survey in the JACC Study baseline questionnaire was used to assess milk, cheese, and yogurt intake. The assessment question was “How frequently do you consume the following items?”. These items included “milk”, “cheese”, and “yogurt”. The available responses were “never”, “1–2 times/month”, “1–2 times/week”, “3–4 times/week”, and “almost every day”. These responses were found to be almost equal to the following amounts: milk: 0.0, 6.4, 26.8, 64.0, and 128 g/day; cheese: 0.0, 0.9, 3.6, 8.5, and 17.0 g/day, and yogurt: 0.0, 4.9, 21.0, 47.0, and 98.0 g/day, respectively.^8^^,^^9^ To obtain statistical power, the frequency responses to “3–4 times/week” and “almost every day” were merged into one group “≥3 times/week”.

We used the Cox proportional hazard regression models to compute the hazard ratios (HRs) and their 95% confidence intervals (CIs) of the incidence of EC for the intakes of different amounts of milk, cheese, and yogurt. Person-years of follow-up were calculated from the date of responding to the JACC Study’s baseline questionnaire to the date of EC diagnosis, death, moving out, or the end of the study, whichever came first.

The results showed that a total of 117 incident cases of EC were diagnosed over a mean follow-up period of 13.0 years. Intakes of milk, cheese, and yogurt were not associated with the risk of EC; the age- and sex-adjusted HRs for the highest versus the lowest intake were 0.81 (95% CI, 0.51–1.30), 0.85 (95% CI, 0.39–1.87), and 0.78 (95% CI, 0.38–1.63), and the multivariable-adjusted HRs were 0.89 (95% CI, 0.55–1.43), 0.77 (95% CI, 0.34–1.71), and 0.82 (95% CI, 0.39–1.73), respectively. The *P*-values for trend across the increasing frequencies of milk, cheese, and yogurt were statistically insignificant. Also, the *P*-values for sex, smoking, and alcohol intake interactions in the three dairy products were statistically insignificant (Table 1).

**Table 1.** The associations between milk, cheese, and yogurt intake and the risk of esophageal cancer incidence among Japanese

	Never	1–2 times/month	1–2 times/week	≥3 times/week	*P* for trend
**Milk**					
Person-years	135,000	60,683	108,155	459,864	—
Total population	10,491	4,627	8,065	35,473	—
Incident cases	24	9	22	61	—
Model I	1	0.82 (0.38–1.77)	1.26 (0.71–2.25)	0.81 (0.51–1.30)	0.805
Model II	1	0.83 (0.39–1.80)	1.29 (0.72–2.31)	0.89 (0.55–1.43)	0.904
**Cheese**					
Person-years	327,950	182,921	98,716	50,613	—
Total population	25,318	13,255	7,007	3,722	—
Incident cases	53	22	17	7	—
Model I	1	0.77 (0.46–1.26)	1.10 (0.64–1.91)	0.85 (0.39–1.87)	0.925
Model II	1	0.74 (0.45–1.23)	1.04 (0.59–1.83)	0.77 (0.34–1.71)	0.732
**Yogurt**					
Person-years	367,641	116,267	84,128	71,809	—
Total population	28,615	9,032	6,555	5,732	—
Incident cases	69	11	2	8	—
Model I	1	0.69 (0.36–1.30)	—	0.78 (0.38–1.63)	0.139
Model II	1	0.72 (0.38–1.37)	—	0.82 (0.39–1.73)	0.182

Model I: Adjusted for age (years) and sex (men and women).

Model II: Adjusted further for body mass index (<25 and ≥25 kg/m^2^), (never smokers, former smoker of <20, former smoker of ≥20, current smoker of <20, and current smoker of ≥20 cigarettes/day), alcohol intake (never, former, and current), walking (never, <30, 30–60, and >60 minutes/day), leisure sports (never, 1–2, 3–4, and ≥5 hours/week), quartiles of daily intakes of meat, vegetables, and total energy (g/day), and family history of cancer (yes and no).

*P*-values for sex interaction (milk = 0.334, cheese = 0.948, and yogurt = 0.339).

*P*-values for smoking interaction (milk = 0.600, cheese = 0.462, and yogurt = 0.974).

*P*-values for alcohol intake interaction (milk = 0.839, cheese = 0.248, and yogurt = 0.692).

Our results are in line with a previous meta-analysis that showed no association between milk intake (pooled estimate from 11 studies: HR 0.93; 95% CI, 0.74–1.16) or cheese intake (pooled estimate from 6 studies: HR 0.84; 95% CI, 0.61–1.15) and the risk of EC. However, the meta-analysis, unlike our results, suggested a weak protective role of yogurt consumption against the risk of EC (pooled estimate from 3 studies: HR 0.73; 95% CI, 0.54–0.98). However, the number of studies investigating yogurt was limited, and significant heterogeneity across the included studies was detected.^10^

This study included several strengths, such as its prospective cohort design; the long follow-up period; using a validated food frequency questionnaire to assess the intakes of milk, cheese, and yogurt; and controlling the results for most sociodemographic, lifestyle, and dietary confounders. However, this study was limited by the small number of incident cases of EC; thus, the insignificant association could be partly due to the reduced statistical power. In conclusion, dairy consumption was not statistically associated with the risk of EC among Japanese. Future large-scale studies are warranted.
